# Unraveling Herpes Zoster Vaccine Hesitancy, Acceptance, and Its Predictors: Insights From a Scoping Review

**DOI:** 10.3389/phrs.2024.1606679

**Published:** 2024-07-24

**Authors:** Xiaolong Wang, Shuhui Shang, Enming Zhang, Zhengyue Dai, Yufei Xing, Jiale Hu, Yaojuan Gao, Qiong Fang

**Affiliations:** ^1^ School of Nursing, Shanghai Jiao Tong University, Shanghai, China; ^2^ College of Health Professions, Virginia Commonwealth University, Richmond, VA, United States; ^3^ Nursing Department, Shanghai Integrated Traditional Chinese and Western Medicine Hospital, Shanghai University of Traditional Chinese Medicine, Shanghai, China

**Keywords:** factors, vaccine hesitancy, acceptance, barriers, herpes zoster

## Abstract

**Objectives:**

Herpes zoster vaccination is critical in preventing herpes zoster virus infection and its associated consequences. Despite its relevance, global herpes zoster immunisation coverage remains alarmingly low. Understanding the factors that drive vaccine scepticism and acceptance is crucial for increasing immunisation rates and improving public health outcomes.

**Methods:**

This scoping review, following Joanna Briggs Institute guidelines, included 18 studies examining vaccine hesitancy, acceptance, and associated factors. Meticulous data analysis revealed hesitancy’s intricate dynamics across countries and demographics.

**Results:**

Studies displayed a wide range of acceptance rates (2.8%–89.02%), showcasing the complex interplay of attitudes and behaviors towards vaccination. Reasons for vaccine refusal were repeatedly identified in this setting, including worries about potential adverse effects, views of vaccine necessity, and vaccine supply constraints. Notably, individuals’ patterns of vaccine acceptance and hesitancy differed among countries, vaccines, and vaccination-related factors.

**Conclusion:**

Addressing acceptance hurdles by improving accessibility, providing accurate information, and strengthening healthcare recommendations is crucial. Understanding the multifaceted factors influencing hesitancy allows for targeted interventions, elevating immunization rates and enhancing public health globally.

## Introduction

Herpes zoster (HZ) is an infectious illness caused by the reactivation of the Varicella-zoster virus [1]. The yearly incidence of HZ spans from 1.2 to 4.8 cases per 1,000 individuals across all age groups, with higher rates of 7.2–11.8 cases per 1,000 individuals among those aged 60 and older [2]. Systemic signs of viral infection include unilateral clusters of skin rashes accompanied by burning sensations and postherpetic neuralgia (PHN), which has a major impact on patients’ quality of life and hence poses a serious public health problem [2].

Vaccines have been critical in the treatment and prevention of a variety of infectious illnesses, including influenza, pneumococcus [3], and COVID-19 [4]. Similarly, the early administration of the Herpes zoster vaccine (HZV) has emerged as the most effective strategy in preventing herpes zoster and lowering the risk of postherpetic neuralgia [5].

However, the lack of awareness about the vaccine [6], inadequate recommendations from healthcare providers [7], financial constraints [8], and limited accessibility [9] may have contributed to a relatively low herpes zoster vaccination rate among the population, fueling the global phenomenon of vaccine hesitancy. Presently, there are two predominant herpes zoster vaccines available globally: Zostavax (zoster vaccine live, ZVL), a single-dose live attenuated vaccine developed by Merck, and Shingrix (recombinant zoster vaccine, RZV), a two-dose recombinant vaccine developed by GlaxoSmithKline. In Canada, an estimated 36.3% of Canadians aged 65 and older had received the shingles vaccine [10]. For US, herpes zoster vaccination coverage (≥1 dose of any type of herpes zoster vaccination) was 32.6% among adults aged 50 and older) [11]. In China, the situation is more challenging, with a mere 3.0% vaccination rate for RZV in 2022 [12]. Confronting this global public health concern pertaining to herpes zoster vaccination, the resurgence of vaccine hesitancy demands urgent attention.

In 2015, the World Health Organization’s Strategic Advisory Group of Experts (SAGE) on Immunization introduced the term “vaccine hesitancy,” characterizing it as a delay in accepting or outright refusal of vaccination notwithstanding the availability of vaccination services. Vaccination hesitancy is a complex phenomenon that varies throughout time periods, geographical regions, and vaccination kinds [13]. Vaccine hesitancy manifests as lower vaccination rates, thereby creating gaps in both individual and communal immunity. This situation paves the way for the recurrence and epidemics. In 2019, the World Health Organization classified vaccine hesitancy as one of the ten threats to global health [14]. The incidence of vaccine hesitancy is even more concerning in the context of COVID-19, where it has been discovered that COVID-19 infection [15] and an imbalance in the body’s immunity caused by vaccination with a specific type of COVID-19 vaccine [16] may contribute to the development of herpes zoster, and that this negative outcome of immunization contributes to an individual’s resistance to subsequent herpes zoster vaccination [17].

Collaborative efforts from the government, society, families, and individuals are essential to address vaccine hesitancy regarding herpes zoster. The government should formulate policies and provide resources, while society enhances public education and awareness. Families should actively participate in decision-making, and individuals should strengthen their health awareness. Collective actions can lead to increased vaccination rates and the protection of public health. In fact, findings from surveys on influenza and COVID-19 vaccines reveal that people’s desire to accept vaccines is heavily impacted by their faith in the government/healthcare institutions [3, 18].

Currently, there is a strong emphasis on etiology, epidemiology, and cross-sectional investigations on vaccination status in the field of herpes zoster vaccine research. Certain crucial aspects remain insufficiently explored, such as effective communication about vaccines and broad-scale governmental/social promotion [19]. In conclusion, vaccine hesitancy and low vaccination willingness are major barriers to achieving low global herpes zoster vaccine coverage. To address this research topic, the present scoping review aims to comprehensively understand the current state of herpes zoster vaccine hesitancy. This includes a thorough assessment of the hesitation levels, influencing factors, and predictive indicators. Furthermore, it actively explores regional variations in vaccination willingness. This will help in identifying major concerns and difficulties associated to vaccine hesitancy, comprehending public attitudes and behaviors toward vaccines, and providing a scientific basis for tailored health policies and interventions for global stakeholders. It will ultimately contribute to the enhancement of herpes zoster prevention and control, hence benefiting public health.

## Methods

### Information Sources

This study followed the methodological guidance outlined in the Joanna Briggs Institute scoping review framework [20]. The primary search terms for the research topic encompassed “herpes zoster vaccine,” “vaccine hesitancy,” “vaccine acceptance willingness,” “vaccination willingness,” “predictive factors,” and related terms. Comprehensive searches were executed across English and Chinese databases, including PubMed, Embase, MEDLINE, Web of Science, CINAHL, China National Knowledge Infrastructure (CNKI), and Wan fang Data Knowledge Service Platform. The search scope extended from the inception of these databases to 30 July 2023.

### Eligibility Criteria

Throughout the literature screening process, a stringent set of inclusion criteria was implemented to ensure the pertinence and caliber of the chosen articles. Firstly, quantitative studies probing herpes zoster vaccine hesitancy, vaccine acceptance, and related predictive factors were encompassed. These investigations spanned various methodological designs, such as randomized controlled trials, cohort studies, and cross-sectional studies, allowing for a comprehensive exploration of the domain. Secondly, for the selected research, rigorous methodological methods and large sample sizes were required. This criterion ensured research dependability and representation, boosting trust in the resulting results. Moreover, the selection was restricted to Chinese or English literature to accommodate readers’ preferences and linguistic capacities, while maintaining linguistic precision. In addition to these criteria, articles with open-access full texts were actively pursued.

In contrast, a set of exclusion criteria was utilized to eliminate literature that was not related to the research topic. This included studies that solely focused on vaccine coverage, clinical etiology, or epidemiological investigation. The primary objective of this study was to ensure the congruence and logical consistency of the research. In addition, papers lacking crucial data or information were excluded, as they were unable to provide the necessary strong evidence needed to support further review analysis. To avoid repetition, duplicate articles or repetitive reports of the same study findings were removed.

**FIGURE 1:**
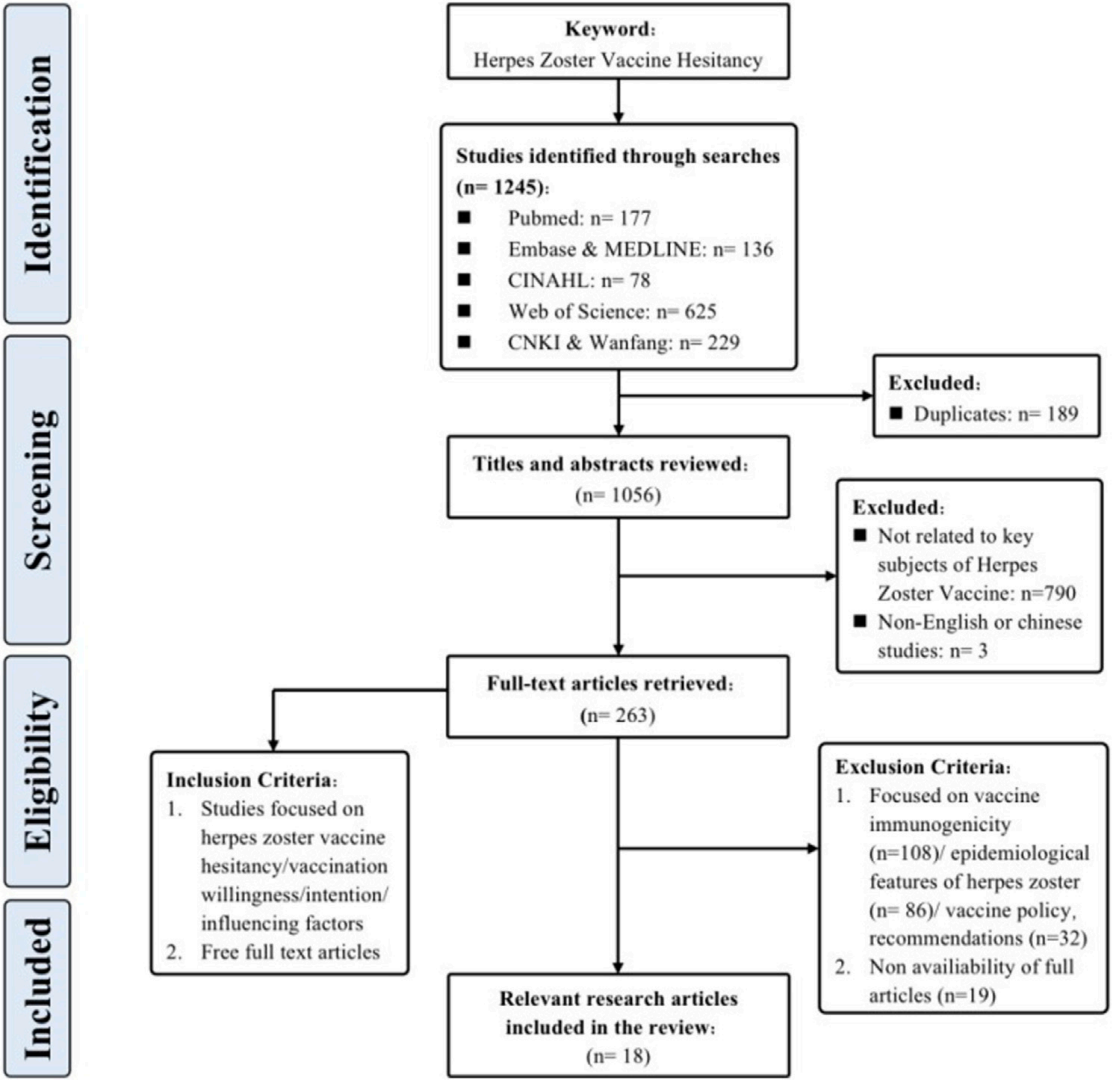
Flowchart of the search strategy and the study selection (China, 2023).

Through meticulous adherence to the specified inclusion and exclusion criteria, a congruent alignment was achieved between the selected literature and our research aims, while also meeting the necessary quality requirements. This has established a strong foundation for subsequent review evaluations. Figure 1 provides a comprehensive portrayal of the screening process.

### Identification and Selection of Reviews

To comprehensively grasp the nuances and core findings of each article, and to establish a robust foundation for subsequent synthesis and analysis, this research diligently executed systematic information extraction and literature selection. The approach employed for literature selection mirrored the selection procedure utilized in prior studies [21], thereby ensuring consistency and comparability. The collected articles, totaling 18 in number, were subjected to a rigorous three-step procedure for the purposes of selection and extraction. During the first phase, bibliographic records obtained from the literature search were integrated into Endnote 20, facilitating the expurgation of duplicates. Subsequently, two researchers (X.L, S.H), who had undergone training in evidence-based courses, independently evaluated the titles and abstracts of the articles, performing a preliminary screening hinging on the alignment with the research focus of herpes zoster vaccine hesitancy. Following this, the other articles underwent a comprehensive examination that involved a detailed analysis of their complete texts. To ensure reliability, inter-rater consistency was maintained through cross-verification. When discrepancies arose throughout the process of selecting literature, a third researcher (E.M/Z.Y) was involved to facilitate discussion and reach a consensus.

### Summary and Synthesis of Results

To gather information from the studies encompassed in the review, authors X.L and S.H collaborated to develop data extraction forms (Supplementary Table S1). Both authors undertook the task of independently inputting the information into the Excel spreadsheet. Subsequently, the compiled data was subsequently subjected to a collaborative evaluation overseen by E.M and Z.Y. The extracted data included the study’s year of publication, author information, geographical scope, article type, research objectives, research design, various characteristics of the population studied (e.g., age distribution, sample size), gender composition, education level, religious affiliation, and geographic coordinates, the methods used to evaluate the study, the indicators used to measure outcomes, and specific information regarding vaccine hesitancy. Q.F supervised and approved the final data extraction form to ensure its precision and applicability.

Both data and visual representations were meticulously generated using GraphPad Prism 9 and Excel 16.7. The subsequent results section summarizes the key findings from the included studies.

### Protocol and Registration

The protocol was filed on 12 August 2023, on the Open Science Framework (OSF) platform.

## Results

### Search Result

In line with the prescribed methodological parameters and procedures, an extensive pool of 1,245 research articles was initially identified, spanning up until 30 July 2023. Employing snowballing technique enabled the identification of supplementary related research articles. Ultimately, a total of 18 research articles that adhered to the inclusion criteria were assimilated into the review. Among the assorted studies included, an overwhelming majority, 17 in number (94.7%), adopted the cross-sectional survey design, whereas a solitary study (5.3%) was classified as a cohort study. The culmination of this process encompassed a grand total of 29,514 participants hailing from 12 different nations, including the United States, Canada, United Kingdom, Netherlands, France, Italy, Turkey, Saudi Arabia, United Arab Emirates, China, South Korea, and Australia.

**FIGURE 2:**
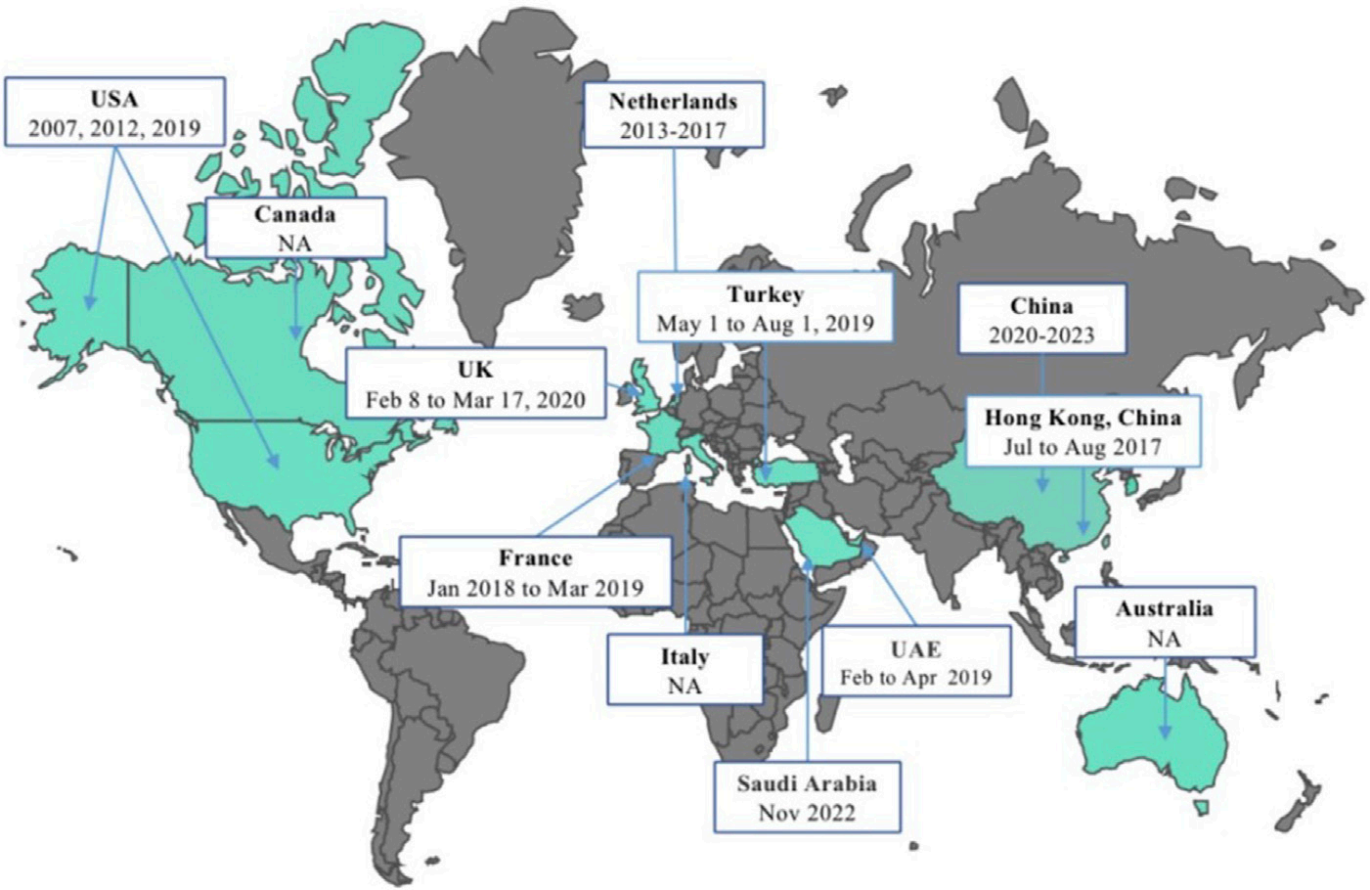
Schedule of the herpes zoster vaccine surveys conducted globally (China, 2023). USA: The United States of America; UK: The United Kingdom of Great Britain and Northern Ireland; UAE: The United Arab Emirates; NA: not applicable.

Figure 2 illustrates the initiation dates or months of vaccine surveys conducted across different regions globally. The first herpes zoster vaccine introduced worldwide was the Zoster Vaccine Live (ZVL), developed by Merck & Co., Inc. This vaccine underwent its initial investigation from May to August 2007 among adults aged 60 and above in the United States. Subsequently, in 2012 and 2019, studies on herpes zoster vaccine acceptance were carried out in the U.S., with the transition from ZVL to the recombinant zoster vaccine (RZV) by GlaxoSmithKline in 2017. In the Americas, Canada initiated a vaccine survey in 2015. Since 2014, several countries in the Western Pacific region, including South Korea, Australia, and China, have engaged in vaccine research endeavors. China stands out for its substantial contribution to studying herpes zoster vaccine hesitancy and acceptance, with five studies included in this research. Two studies were done in the Mediterranean region by the United Arab Emirates and Saudi Arabia between the periods of February–April 2019 and November 2022, respectively. Among European nations, France conducted a nationwide survey from January 2018 to March 2019. Subsequently, the UK conducted a vaccine survey focusing on healthcare worker recommendations in 2021, marking a significant vaccine acceptance assessment since the UK’s vaccination program implementation in 2013. In the Netherlands, during the sixth wave of a nationwide population-based cohort study spanning 2013 to 2017, inquiries regarding vaccination acceptability and infectious diseases were incorporated, specifically addressing herpes zoster vaccine acceptance.

### Variables Examined in Herpes Zoster Surveys

#### Study Variables in General

As shown in Table 1, all studies on herpes zoster vaccines (*n* = 18, 100%) assessed demographic characteristics. Chronic disease history, knowledge level regarding herpes zoster disease and its vaccine, history of chickenpox and herpes zoster infection, vaccination history, vaccination recommendations by healthcare providers, and other such variables were commonly assessed in herpes zoster vaccine surveys. Vaccine attitudes (vaccine hesitancy (*n* = 4, 21%), vaccine acceptance (*n* = 14, 74%), intention (*n* = 6, 32%) and analysis of barriers to vaccination (*n* = 9, 47%) have emerged as key themes in recent years’ research. Several studies also documented variables such as trust in government and immunization institutions, individual and collective responsibility, and vaccine accessibility.

**TABLE 1:** Variables examined in the herpes zoster survey study (China, 2023).

Study variables	Number of studies	Study ID
Sociodemographic	18 (100%)	[1–18]
Chronic disease	10 (53%)	[1], [3–5], [7–11] [18]
Knowledge of HZ	12 (63%)	[1–4], [6, 7], [9–11], [13, 14, 16]
Knowledge of HZV	11 (58%)	[1, 3, 4, 6, 7], [9–11], [13, 14, 16]
History of HZ infection	10 (53%)	[2], [4–9], [12, 15, 18]
History of chickenpox infection	6 (32%)	[4, 7, 9, 10, 12, 15]
HCW recommendations	10 (53%)	[4, 8, 9], [11–17]
Vaccination history/rate	11 (58%)	[1, 2, 5], [7–13], [15, 16]
Trust in authorities and policy support	8 (42%)	[4, 5, 8, 9, 12, 14, 16, 17]
Risk perception of HZ	9 (47%)	[1], [3–7], [12, 13, 15]
Vaccine hesitancy	4 (21%)	[3, 5, 12, 16]
Vaccine willingness/acceptance	14 (74%)	[1–3], [5–9], [12, 13], [15–18]
Vaccine intention/attitude	6 (32%)	[4, 5], [10–12], [14]
Reasons of not accepting vaccine	9 (47%)	[1, 4, 5], [10–15]

#### General Population Herpes Zoster Vaccine Acceptance, Intention, and Hesitancy Worldwide

**FIGURE 3:**
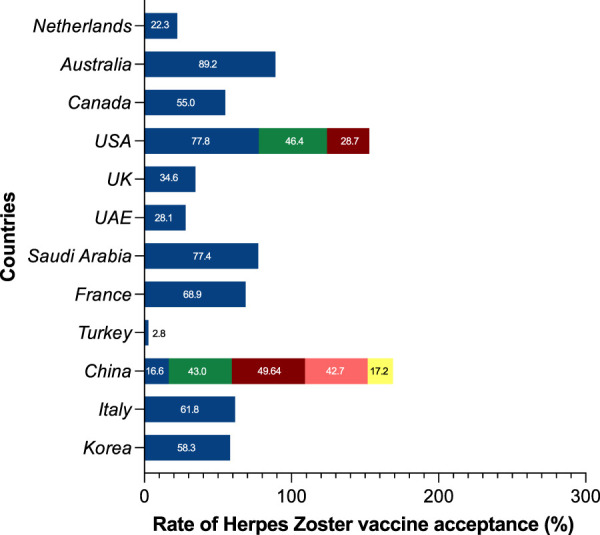
Global prevalence of herpes zoster vaccine acceptance, intention, and hesitancy (China, 2023). USA: The United States of America; UK: The United Kingdom of Great Britain and Northern Ireland; UAE: The United Arab Emirates.

Among the 18 studies included in this analysis of herpes zoster vaccines, the examined themes encompassed vaccine hesitancy, vaccine acceptance or intent, and vaccine attitudes. It is worth noting that a significant proportion of these investigations were conducted in the United States and China. Several nations, including Australia (89.2%), the United States (77.8%), Saudi Arabia (77.4%), France (68.9%), Italy (61.8%), and South Korea (58.3%), demonstrated higher percentages of acceptability. In contrast, the United Kingdom exhibited a herpes zoster vaccination intention of 34.6%, while the United Arab Emirates and China displayed lesser intentions at 28.1% and 42.3%, respectively. It’s important to highlight that within the three U.S. studies included, the change in national immunization policy led to a movement from the recommendation of the herpes zoster vaccine live to the recombinant zoster vaccine after 2017. Notably, findings pertaining to the intention to use RZV revealed vaccination rates below 50% (Figure 3).

#### Difference Between Attitude and Behavior

**FIGURE 4:**
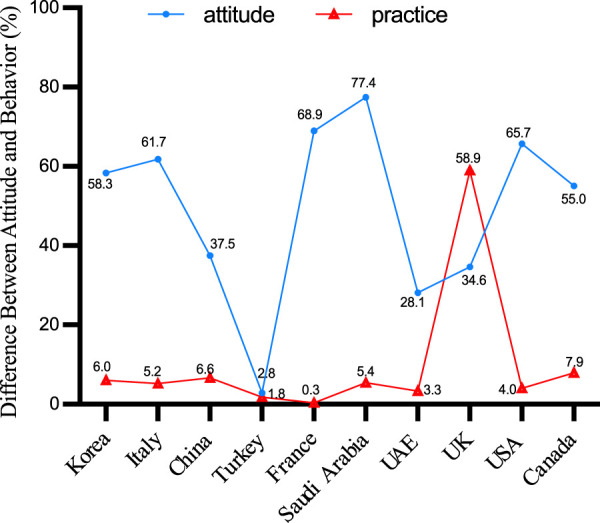
Gap in willingness to vaccinate and vaccination coverage (China, 2023).

Across the ten countries examined, a disparity between vaccine intent and actual vaccination rates is apparent, often reflecting a pattern of high intent but limited regional coverage. Except for Turkey, both intent and actual vaccination rates remained below 3%. Remarkably, UK displayed a lower level of intention to vaccinate, despite the comparatively high coverage of the herpes zoster vaccination. The graphical analysis did not include the data points from two Australian and Dutch studies included in the evaluation due to the lack of reported coverage rates (Figure 4).

The contrast between attitudes and behaviors has consistently been a pivotal aspect of vaccine hesitancy research. As per the World Health Organization’s delineation of vaccine hesitancy, this phenomenon resides within the spectrum that spans from full acceptance (high demand for vaccines) to complete rejection (no vaccine demand) and represents a behavioral manifestation. However, this assertion may not be sufficiently rigors in the current research context. Some researchers contend that vaccine hesitancy reflects a person’s propensity towards vaccination. In practice, however, they are frequently indistinguishable. For the question of herpes zoster vaccine immunization schedules, relying merely on the percentage of unvaccinated individuals may not capture the concept of vaccine hesitancy adequately.

### Key Factors of the Included Studies

The phenomenon of vaccine hesitancy is inherently intricate and contingent upon various contextual circumstances [22]. To actively guide appropriate herpes zoster vaccine uptake globally, enhance vaccine intent, and reduce vaccine hesitancy, it is essential to delineate the influencing factors of vaccine hesitancy. This section aims to incorporate the Vaccine Hesitancy Determinants Matrix proposed by the SAGE working group on vaccine hesitancy and summarize the influencing factors involved in 18 studies (Table 2).

**TABLE 2:** Influencing factors via vaccine hesitancy determinants matrix (China, 2023).

Framework	Factors
Individuals/groups	a. Socio-demographic factors: age gender education level monthly income nationality/area careers b. Vaccination experience of individuals, families and/or community members: chronic diease history of chickenpox infection/herpes zoster infection vaccination experience c. Perceived risk/benefit
Social environment	a. Media communication environment
	b. Religion/Culture/Socioeconomic
	c. Geographical barriers
Vaccines/vaccination	a. Vaccine accessibility/affordability b. Level of confidence in vaccine safety and efficacy c. Influenza vaccination d. Strength of recommendations and/or knowledge base and/or attitudes of healthcare professionals

#### Individual/Group Factors

Among the included 18 studies, factors such as age, educational level, residence, gender, income level, region, presence of chronic diseases, history of varicella/herpes zoster infection, and herpes zoster vaccine immunization history were commonly reported. Due to varying national vaccination policies, the age ranges in the studies varied, including 19 years, 50 years and older, 60 years and older, 65 years and older, etc. Four studies (22.2%) found a significant association between age and vaccine uptake [23–26]. Higher levels of education were associated with greater vaccine intent in four studies (22.2%) [23]. Moreover, there was a notable correlation between gender and intention in six studies (33.3%). Notably, investigations conducted in China [27], Saudi Arabia [28], the United States [29], and the Netherlands [30] revealed a greater inclination towards vaccine acceptance among females, while French research suggested a higher probability of vaccine adoption among males [31]. Residents with higher family income demonstrated more favorable attitudes and stronger intent toward herpes zoster vaccine uptake [27]. Regarding personal, family, and/or community vaccination experiences, while five studies included chronic diseases as a variable, no significant results clearly indicated its association with vaccine intent. However, four studies demonstrated that individuals with a clear risk perception of herpes zoster disease and good knowledge of herpes zoster vaccines, particularly those who had experienced the infection themselves or had close acquaintances with a history of infection, had a greater vaccine intent [31–34]. In terms of vaccine awareness/knowledge, nearly 12 studies (66.7%) described evaluations of residents’ knowledge about herpes zoster disease and vaccines. However, in multifactor regression analyses, only 6 studies indicated that possessing knowledge about the disease and vaccine was conducive to promoting vaccine intent.

#### Social Environment Factors

The advent of social media has significantly facilitated the dissemination of information. Predictably, those who oppose vaccination have also utilized online platforms to spread their views. In contrast to conventional communication channels, social media platforms offer a novel means of disseminating information, but they have also emerged as a unique channel for the dissemination of anti-vaccination content. A study found that the spread of anti-vaccine content, vaccine misinformation, “conspiracy theories,” and associated content on social media platforms has had significant negative effects on the public health landscape [23]. Besides, a greater proportion of residents and medical professionals rely on online sources for health-related information than the general population. This trend has had a significant impact on vaccination decisions, primarily as a result of the variable quality of vaccine-related content available on websites and social media platforms [27]. Addressing vaccine hesitancy is a potentially viable and effective solution to this problem.

In the realm of social environmental factors, previous research on vaccinations has indicated that elements such as religious convictions and cultural heritage can potentially exert an influence on the phenomenon of vaccine hesitancy. Nevertheless, it is worth noting that the characteristics may not have been comprehensively examined in the assessment of the incorporated research. Consequently, a more in-depth analysis of these issues will be addressed afterwards. Therefore, it will be added in the discussion section later.

#### Vaccines/Vaccination Factors

The administration of the herpes zoster vaccine is influenced by various factors, including the accessibility, price, and desire to pay for the vaccine. A study from Hong Kong [35] suggested that the influence of knowledge and awareness on herpes zoster appears to be less important compared to the prominent role played by the high cost of the vaccine.

In a survey of 50-year-olds’ propensity to receive the herpes zoster vaccine, Qiu discovered that the primary reason for refusal was the “high cost of the vaccine.” The herpes zoster vaccine consists of two doses, each costing around 1,600 yuan (about $220), which posed a financial burden for middle-aged and elderly individuals [30]. Additionally, three studies observe a significant positive correlation between the level of trust in vaccine safety and efficacy and vaccination willingness [36]. A study conducted in the United Kingdom reveals that 14.2% of respondents exhibited vaccine hesitancy, expressing doubts about vaccine effectiveness and perceiving inadequate vaccine safety assurance from local healthcare institutions [37]. This lack of confidence in the relevant vaccination program contributes to a higher likelihood of vaccine non-acceptance.

Furthermore, three studies report the influence of influenza vaccine uptake on herpes zoster vaccine willingness [34]. Additionally, healthcare professionals’ vaccine recommendations are positively associated with a higher willingness to receive the herpes zoster vaccine. Seven studies from six countries (China, the Netherlands, Canada, the United States, the United Kingdom, and the United Arab Emirates) indicate that vaccine recommendations contribute to strengthening participants’ willingness to get vaccinated.

### Barriers to Vaccination

As illustrated in Supplementary Figure S1, the final segment of this study delved into an analysis of the primary factors contributing to vaccine refusal. Nine studies (50.0%) from seven different countries—South Korea, China, the United Kingdom, Saudi Arabia, the United Arab Emirates, and the United States—provided insights into the key barriers to vaccine acceptance. Inadequate access to vaccines was found as the primary obstacle in this investigation, accounting for 35.11% of the stated reasons for rejection [38]. Concerns about vaccination side effects were closely followed, accounting for 32.76% of the reported barriers [39]. The notion of good personal health, which leads to a lack of need for vaccination, came in third (26.71%) [22, 32]. In addition, insufficient awareness about herpes zoster and the associated vaccine, as well as a dearth of recommendations from healthcare professionals, were also noteworthy reasons, accounting for 24.67% of the cited barriers.

## Discussion

Over the past few years, there has been a progressive rise in the prevalence of herpes zoster. The anticipated rise in life expectancy is projected to contribute to a corresponding increase in the occurrence of postherpetic neuralgia (PHN), thereby amplifying the burden on public health [40]. The herpes zoster vaccine has emerged as a significant component of adult vaccination and immunization initiatives in certain Western nations, and there is still great potential for its global popularization [41]. Six studies from four countries explored Vaccine intention/attitude, ten studies solely assessed vaccine willingness, and four studies combined vaccine hesitancy evaluation with assessment of vaccine acceptability among surveyed populations. Seven studies aimed at adults aged 50 and older were closely aligned with country-specific vaccination recommendations. Recommendations for vaccine age eligibility differ: Austria, Canada, and the United States recommend RZV vaccine for those aged 50; the Netherlands, and the United Kingdom recommend 60; and Italy and Spain recommend 65 or older.

In the analysis of sociodemographic factors, educational level emerged as a pivotal influence on awareness across all studies, underscoring the significance of education in health communication and education [41, 42]. Providing comprehensive and reliable information about diseases and their prevention to residents is imperative to mitigate misconceptions and false dissemination. A study from Canada found that the herpes zoster vaccine uptake rates for males and females were 4.8% and 7.3%, respectively [43]. The vaccination rate was higher among urban residents (6.2%) than among those living in rural areas (5.3%) [44]. This indicates that population structure also influences vaccine uptake behavior. Additionally, individuals who have personally experienced relevant diseases or who have family/relatives/friends with vaccination experience are less likely to exhibit vaccine hesitancy [45]. Personal experience with the disease or exposure to vaccinated individuals tends to enhance the understanding of vaccine importance and benefits, leading to a more positive and willing attitude towards vaccination when confronted with the decision [35]. This direct exposure and experience provide tangible evidence and personal insight, which can strengthen vaccine awareness and confidence. Herpes zoster is more likely to develop in people with chronic diseases or comorbidities [46]. On average, individuals with at least one of the following conditions: asthma, chronic heart disease, chronic obstructive pulmonary disease (COPD), depression, and rheumatoid arthritis, have a 30% increased risk of acute herpes zoster [47]. Over two-thirds of individuals who had experienced herpes zoster themselves or had family members or friends with herpes zoster recognized the significant burden of pain and rash, valued prevention, and were more likely to proactively receive the vaccine [1].

Since 2007, a growing corpus of research has been conducted on the willingness of individuals to receive the herpes zoster vaccine. The introduction of the RZV vaccine further intensified research interest in herpes zoster. Australia consistently demonstrates the highest vaccine acceptance rate, followed by the United States, Saudi Arabia, and France, among others. In comparison to China, residents of most foreign regions are more inclined to receive the herpes zoster vaccine. However, high vaccine willingness does not necessarily translate to high regional vaccine coverage rates. Except for the region of Turkey, both willingness and actual coverage rates remain below 3%. Data from the majority of countries indicate that respondents’ willingness to be vaccinated has increased, but the actual vaccination rate remains low. It’s noteworthy that even in developed countries, there exists significant variability in herpes zoster vaccine uptake rates. This variation is strongly correlated with the approval date of the herpes zoster vaccine, with countries that introduced the HZ vaccine earlier exhibiting higher vaccination rates.

In 2007, only 1.9% of 3,662 people aged 60 or older in the United States reported receiving the herpes zoster vaccine. 78% of those who were unaware of the herpes zoster vaccine said they would contemplate getting it if their doctor recommended it [23]. Since 2008, when the U.S. Centers for Disease Control and Prevention Advisory Committee on Immunization Practices recommended a single dose of the herpes zoster vaccine for individuals aged 60 and older, herpes zoster vaccination rates in various U.S. states have exhibited a consistent upward trend [48]. In 2014, the rate was 31.8%; by 2017, it had increased to 34.9%, surpassing the 2020 target rate of 30% for healthy individuals [49].

Regarding vaccine willingness, the public’s trust in government and healthcare institutions has a direct impact on their attitudes and acceptance of vaccines, especially in terms of accessing and comprehending vaccine-related information [50]. When the public has a high level of trust in these organizations, they are more likely to embrace vaccine recommendations and information from them, resulting in increased vaccination rates. Governments and medical institutions should enhance transparency and communication, establish public participation mechanisms, carefully consider public opinions and proposals, and increase public participation and confidence. This will help overcome vaccine information barriers, improve public comprehension and acceptance of vaccines, and ultimately increase vaccination willingness [51].

There is also a significant correlation between interactions on social media and public skepticism about vaccine safety [52]. In particular, the study found a substantial link between the spread of disinformation and declines in vaccination rates. The impact of disinformation was particularly pronounced in middle-developed countries, where it led to an increase of approximately 15% in the number of negative tweets about vaccinations [53]. This phenomenon highlights the importance of information regulation on social media platforms and points to the need to counteract the spread of disinformation through public education and correct information dissemination [54].

Distinguishing between Herpes zoster vaccine hesitancy and general vaccine hesitancy more generally is critical to understanding specific vaccination challenges. One is the audience population, where studies in COVID-19 found that the older the age, the lower the probability of vaccine hesitancy [50, 55], a trend that has also been demonstrated in studies of herpes zoster vaccine [12]. Whereas the risk of herpes zoster occurrence itself is more skewed towards people aged 50 years and older, with a clear problem of decreasing age of onset and long duration of illness compared to other preventable disease types, younger people often perceive themselves to be in good health in terms of vaccination, and this has broadly impacted on their willingness to be vaccinated [56].

Secondly, the price of the vaccine, unlike routine vaccines that are free or covered by health insurance, the herpes zoster vaccine is currently available free of charge in a few countries [57] and remains voluntary in most areas. Income constraints make it even more important for populations to weigh the economic benefits of vaccination. Lu et al. found that the willingness to receive herpes zoster vaccine varied the most under different payment scenarios, and the proportion of those willing to receive the vaccine under the health insurance payment scenario was about 4.5 times higher than that under the out-of-pocket payment scenario, which may be related to the higher price of the vaccine and the fact that it is currently a non-immunization vaccine [58]. Public trust in and acceptance of the vaccine can be further enhanced by increasing public awareness of the herpes zoster vaccine and by lowering the financial barriers to vaccination through policy and economic incentives. This requires not only the efforts of the government and healthcare providers, but also the broad participation and support of all sectors of the community.

One limitation of our scoping review is that with the exception of the longitudinal study conducted in the Netherlands, the studies were mostly cross-sectional and the age groups of the examined populations were not fully standardized, so it is unclear how representative the samples of the included studies were. Even though sampling techniques did not reveal any significant differences, caution is still required when interpreting the results. Due to the late introduction of the recombinant herpes zoster vaccine, some middle- and low-income nations have not conducted extensive research on the subject, which may have led to unreported influencing factors. Future research should be conducted in depth to expand and refine the scope. In the final analysis, only English and Chinese literature were included.

The study has certain implications. Firstly, the literature synthesis using the scope review framework offers a comprehensive understanding of the multiple factors that influence individual vaccination behavior against herpes zoster. This comprehensive perspective serves as a valuable resource for researchers exploring this area. Secondly, by gaining insights into individual hesitancy and acceptance of the herpes zoster vaccine, healthcare providers can tailor their educational and communication strategies to effectively increase vaccination willingness. Finally, the study identifies key barriers to vaccination, which can guide health policymakers in rationalizing resource allocation to address issues such as vaccine accessibility and public awareness, ultimately contributing to improved public health outcomes.

### Conclusion

This study provides a comprehensive analysis of the literature regarding herpes zoster vaccine hesitancy, vaccine willingness, and the predictive factors associated with them, based on a scoping review framework. The acceptance rates, intention rates, and hesitancy rates for herpes zoster vaccines exhibit significant variations on a global scale. Challenges in vaccine accessibility, concerns about potential side effects, insufficient awareness of the disease and its vaccine, and limited healthcare provider recommendations are key barriers to vaccination. While studies have cast light on the multifaceted factors that influence vaccine hesitancy among individuals, a more comprehensive analysis encompassing personal, social, and macro-level policies is still required.
