# Supplementary material for: Unraveling Herpes Zoster Vaccine Hesitancy, Acceptance, and Its Predictors: Insights From a Scoping Review
Source: Public Health Rev. 2024 Jul 24;45:1606679. doi: 10.3389/phrs.2024.1606679 (PMC11303218; doi:10.3389/phrs.2024.1606679)
Supplement: Supplementary file 1 [file DataSheet1.docx]

**1 Supplementary Table**

**Table S1:** Characteristics of 18 included studies (China, 2023).

| **ID** | **Year** | **Country** | **Study Design** | **N** | **Population** | **Vaccination Rate** | **Vaccination Topic** | **Rreference** |
| --- | --- | --- | --- | --- | --- | --- | --- | --- |
| 1 | 2015 | Korea | Cross-sectional survey | 607 | ≥50 | 6% | Acceptance | (Roh et al., 2015) |
| 2 | 2010 | Italy | Cross-sectional survey | 3173 | ≤20, 21-40, ≥41 | 5.2% | Willingness | (Parlato et al., 2010) |
| 3 | 2020 | China | Cross-sectional survey | 1672 | 50-69 | NA | Willingness | (Lu X et al., 2021) |
| 4 | 2017 | HK, China | Cross-sectional survey | 408 | ≥50 | NA | Attitude | (Lam et al., 2017) |
| 5 | 2022 | China | Cross-sectional survey | 3838 | ≥50 | 10.34% | Hesitancy | (Jiang et al., 2022) |
| 6 | 2021 | China | Cross-sectional survey | 562 | ≥50 | NA | Willingness | (Qiu et al., 2021) |
| 7 | 2023 | China | Cross-sectional survey | 2864 | ≥25 | 1.7% (≥50) | Willingness | (Wang et al., 2023) |
| 8 | 2019 | Turkey | Cross-sectional survey | 326 | ≥65 | 1.8% | Intention | (Kizmaz et al., 2020) |
| 9 | 2020 | France | Cross-sectional survey | 907 | ≥65 | 0.33% | Acceptance | (Del Signore et al., 2020) |
| 10 | 2023 | Saudi Arabia | Cross-sectional survey | 500 | ≥50 | 5.4% | Willingness | (Alhothali et al., 2023) |
| 11 | 2022 | UAE | Cross-sectional survey | 420 | ≥50 | 3.3% | Hesitancy | (Al-Khalidi et al., 2022) |
| 12 | 2021 | UK | Cross-sectional survey | 372 | ≥65 | 58.90% | Hesitancy | (Nicholls et al., 2021) |
| 13 | 2007 | USA | Cross-sectional survey | 3662 | ≥60 | 1.90% | Willingness | (Lu P et al., 2009) |
| 14 | 2019 | USA | Cross-sectional survey | 381 | ≥50 | NA | Intention | (Baalbaki et al., 2019) |
| 15 | 2012 | USA | Cross-sectional survey | 1000 | ≥50 | 11.90% | Acceptance | (Javed et al., 2012) |
| 16 | 2015 | Canada | Cross-sectional survey | 4023 | Adults | 7.90% | Willingness | (MacDougall et al., 2015) |
| 17 | 2014 | Australia | Cross-sectional survey | 1330 | ≥60 | NA | Willingness | (Litt et al., 2014) |
| 18 | 2023 | Netherlands | Cohort Study | 2933 | 46～86 | NA | Willingness | (Maertzdorf et al., 2023) |

**2 Supplementary Figure**

**Figure S1:** Vaccination status and top reasons of vaccine resistance (China, 2023).

**3 Literature search strategy for PubMed**

#1 ((Herpes Zoster Vaccine [Title/Abstract]) OR (Zoster Vaccine [Title/Abstract]) OR (Shingles Vaccine [Title/Abstract]))

#2 ((Vaccine Hesitancy [Title/Abstract]) OR (Attitude* [Title/Abstract]) OR (Willingness [Title/Abstract]) OR (Acceptance [Title/Abstract]))

#3 ((Research [Title/Abstract]) OR (Survey* [Title/Abstract]) OR (Questionnaire* [Title/Abstract]))

#4 (#1 AND #2) AND #3
